# Supplementary material for: Reduced secretion of neuronal growth regulator 1 contributes to impaired adipose-neuronal crosstalk in obesity
Source: Nat Commun. 2022 Nov 25;13:7269. doi: 10.1038/s41467-022-34846-w (PMC9700863; doi:10.1038/s41467-022-34846-w)

Reduced secretion of neuronal growth regulator 1 contributes to impaired adipose-neuronal crosstalk in obesity

Elisa Duregotti[1], Christina M. Reumiller[1], Ursula Mayr[1], Maria Hasman[1], Lukas E. Schmidt[1], Sean A. Burnap[1], Konstantinos Theofilatos[1], Javier Barallobre-Barreiro[1], Arne Beran[2], Maria Grandoch[2], Alessandro Viviano[1,3,4], Marjan Jahangiri[4] and Manuel Mayr[1*].

1. King's College London British Heart Foundation Centre, School of Cardiovascular Medicine and Sciences, London, United Kingdom. *email: manuel.mayr@kcl.ac.uk
2. Institute of Translational Pharmacology, University Hospital Düsseldorf, Heinrich-Heine-University Düsseldorf, Germany.
3. Cardiothoracic Surgery, Waikato District Health Board, Hamilton, New Zealand.
4. Department of Cardiothoracic Surgery, St. George's Hospital, University of London, London, United Kingdom.

Supplementary Figure 1

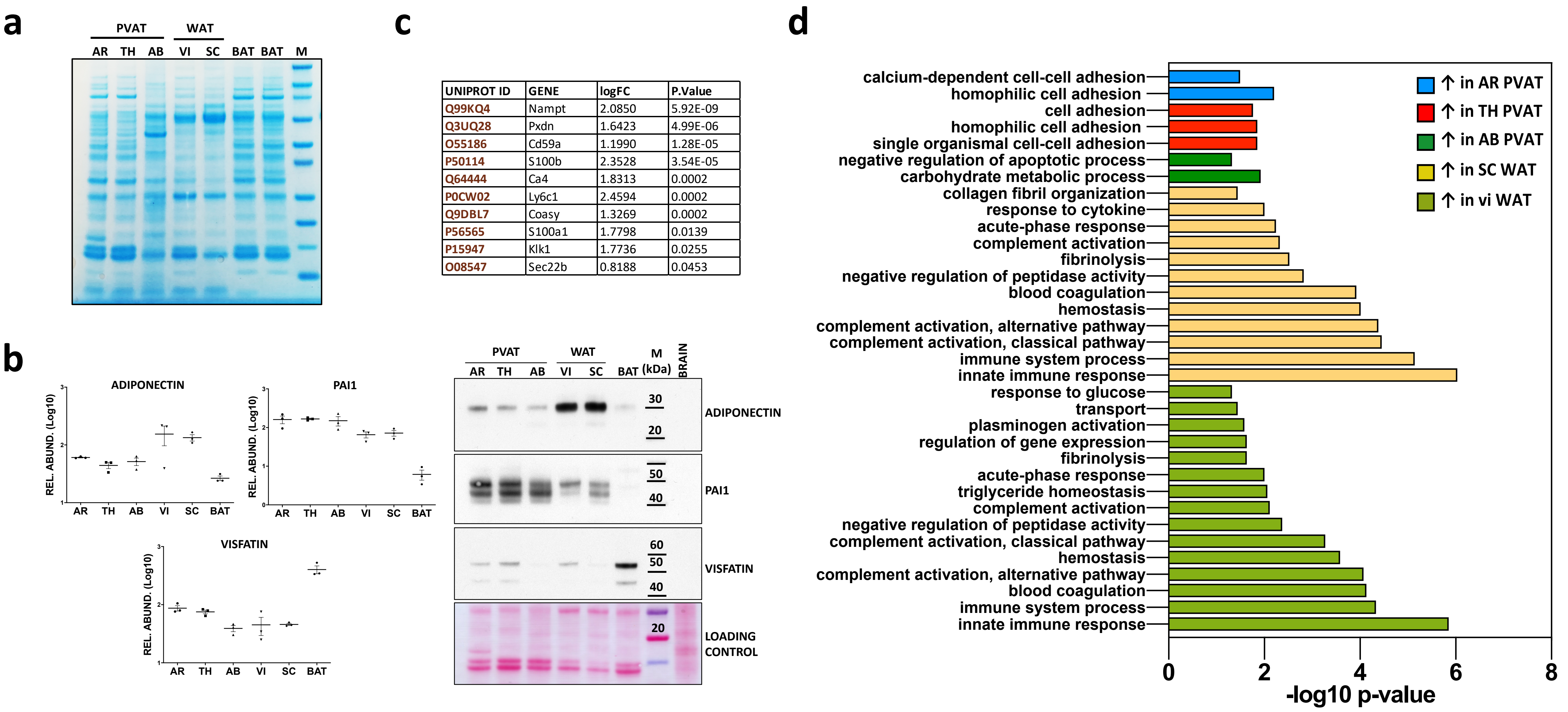

UNIPROT ID	GENE	logFC	P.Value
Q99KQ4	Nampt	2.0850	5.92E-09
Q3UQ28	Pxdn	1.6423	4.99E-06
O55186	Cd59a	1.1990	1.28E-05
P50114	S100b	2.3528	3.54E-05
Q64444	Ca4	1.8313	0.0002
P0CW02	Ly6c1	2.4594	0.0002
Q9DBL7	Coasy	1.3269	0.0002
P56565	S100a1	1.7798	0.0139
P15947	Klk1	1.7736	0.0255
O08547	Sec22b	0.8188	0.0453

Supplementary Figure 1. Proteomics analysis on AT conditioned media. a, Coomassie-stained gel of representative AT conditioned media samples. **b,** MS-detected relative abundances of selected adipokines and validation by Western blot on pooled (N=3) conditioned media samples. Scatter dot plots show mean ± SEM. Source data are provided as a Source Data file. **c,** Table showing proteins significantly upregulated in the secretome of BAT when compared to the other 5 ATs. The limma package was used to compare different groups using the Ebayes algorithm (paired analysis) followed by Benjamini-Hochberg adjustment. **d,** Gene ontology analysis showing the Biological Processes enriched in distinct AT depots secretomes (paired analysis). Fisher's Exact test p-values are shown.

AR: aortic arch PVAT; TH: thoracic PVAT; AB: abdominal PVAT; VI: visceral and SC: subcutaneous white AT (WAT); BAT: interscapular brown AT. UCP1: uncoupling protein 1. PAI1: plasminogen activator inhibitor 1. M: molecular weight marker. Source data are provided as a Source Data file.

Supplementary Figure 2

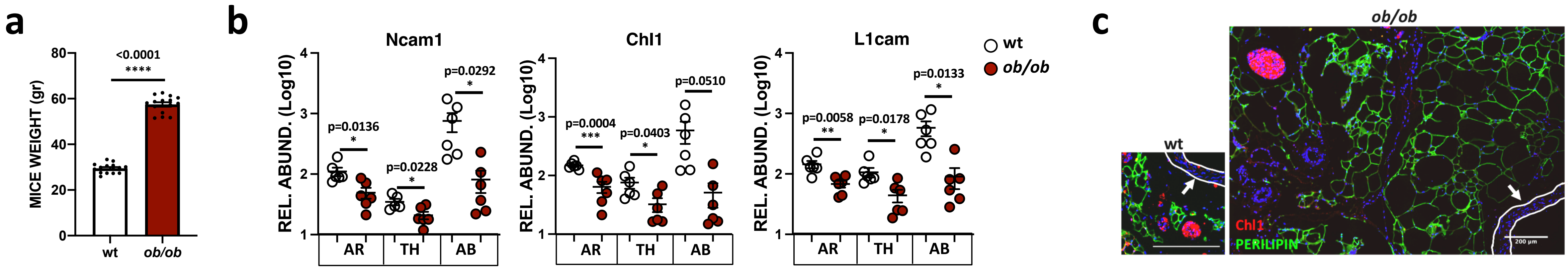

Supplementary Figure 2. Neuronal cell-adhesion molecules in the secretomes of *ob/ob* PVAT depots. a, Body weight of wt and *ob/ob* mice at 18 weeks of age. N=15 mice per group, mean ± SEM, unpaired, two-tailed, independent t-test (p=7.07E-21). **b,** MS-determined relative abundances of cell-adhesion molecules Ncam1, Chl1 and L1cam in distinct wt and *ob/ob* PVAT depots secretomes. N=6 biological replicates per group, unpaired, two-tailed, independent t-test. Mean ± SEM are shown. **c,** Representative immunofluorescence images of wt and *ob/ob* AB PVAT sections stained for perilipin and Chl1. Nuclei are counterstained with DAPI. White arrows point at the aortic wall. Scale bar: 200 µm in both pictures.

AR PVAT: aortic arch perivascular AT. TH PVAT: thoracic PVAT. AB PVAT: abdominal PVAT. Ncam1: neural cell-adhesion molecule 1; Chl1: close homologue of L1; L1cam: neural cell-adhesion molecule L1. Source data are provided as a Source Data file.

Supplementary Figure 3

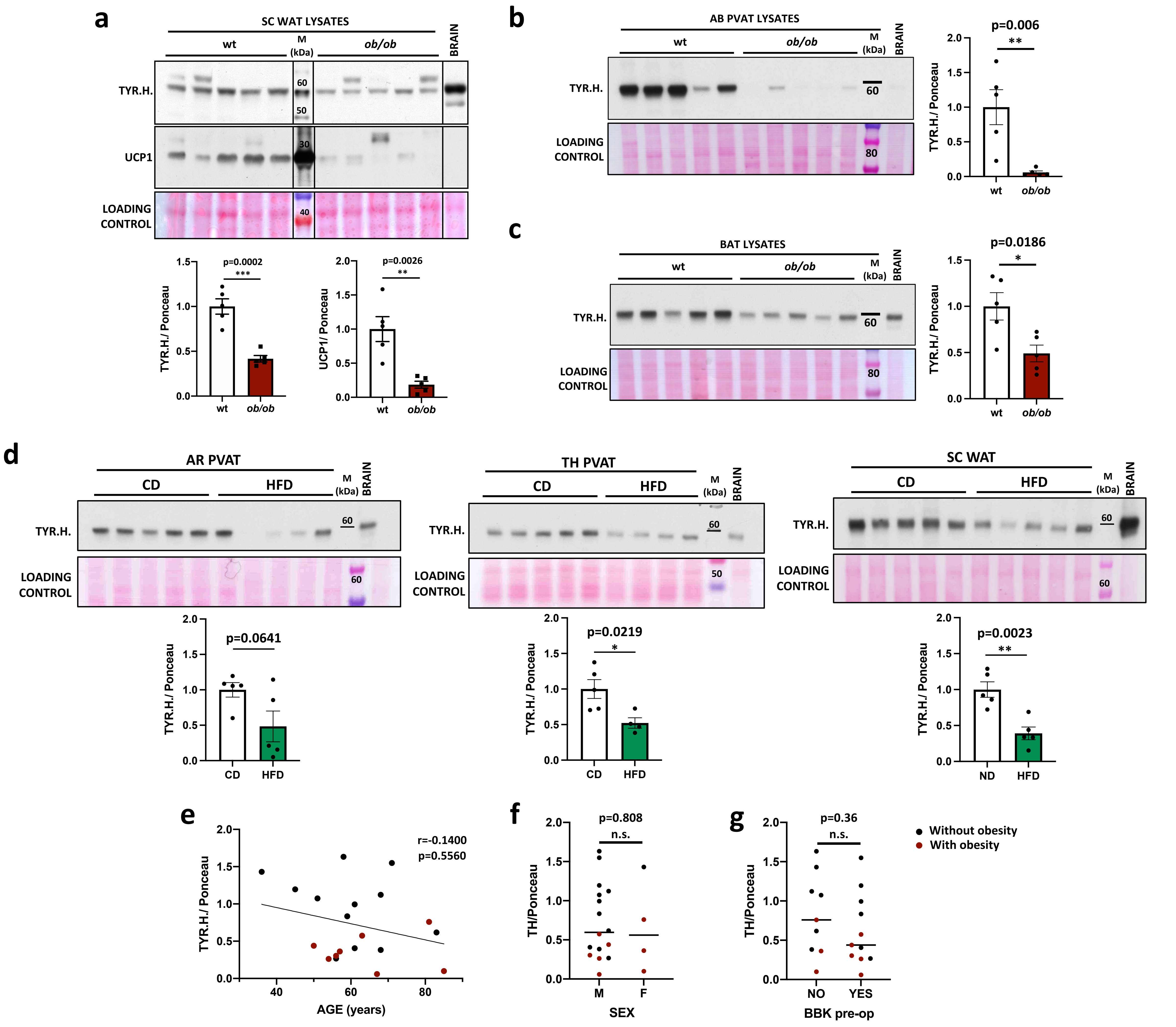

Supplementary Figure 3. Obesity-induced alterations in murine and human AT innervation. **a,** Western blot showing TYR.H. and UCP1 abundances in wt and *ob/ob* SC WAT lysates and relative quantifications. Data are shown as mean ± SEM, N=5 biological replicates, unpaired, two-tailed, independent t-test. **b** and **c**, Western blot showing TYR.H. abundancies in wt and *ob/ob* AB PVAT (**b**) and BAT (**c**) lysates and relative quantifications. Data are shown as mean ± SEM, N=5 biological replicates, unpaired, two-tailed, independent t-test. **d**, Western blot and relative quantifications showing TYR.H. abundances in AR and TH PVAT and SC WAT of wt mice fed with a high-fat diet (HFD) or a control diet (CD) for 16 weeks. Data are shown as mean ± SEM, N=5 biological replicates per group except the HFD TH PVAT group (N=4), and analyzed by unpaired, two-tailed, independent t-test. **e**, Linear regression analysis showing the correlation between

TYR.H. levels assessed by western blot and age. N=20, Spearman correlation analysis, two-tailed p-value. **f** and **g**, Scatter dot plots showing the levels of TYR.H. in the epicardial fat of human patients grouped according to their sex (**f**) and pre-op administration of β-blockers (**g**). N=20 study participants, 12 non-affected and 8 affected by obesity, median is shown, unpaired, two-tailed, independent t-test.

SC WAT: subcutaneous white AT. AB: abdominal PVAT; BAT: interscapular brown AT. TYR.H.: tyrosine hydroxylase. UCP1: uncoupling protein 1. BBK: beta blockers. M: molecular weight marker. Source data are provided as a Source Data file.

Supplementary Figure 4

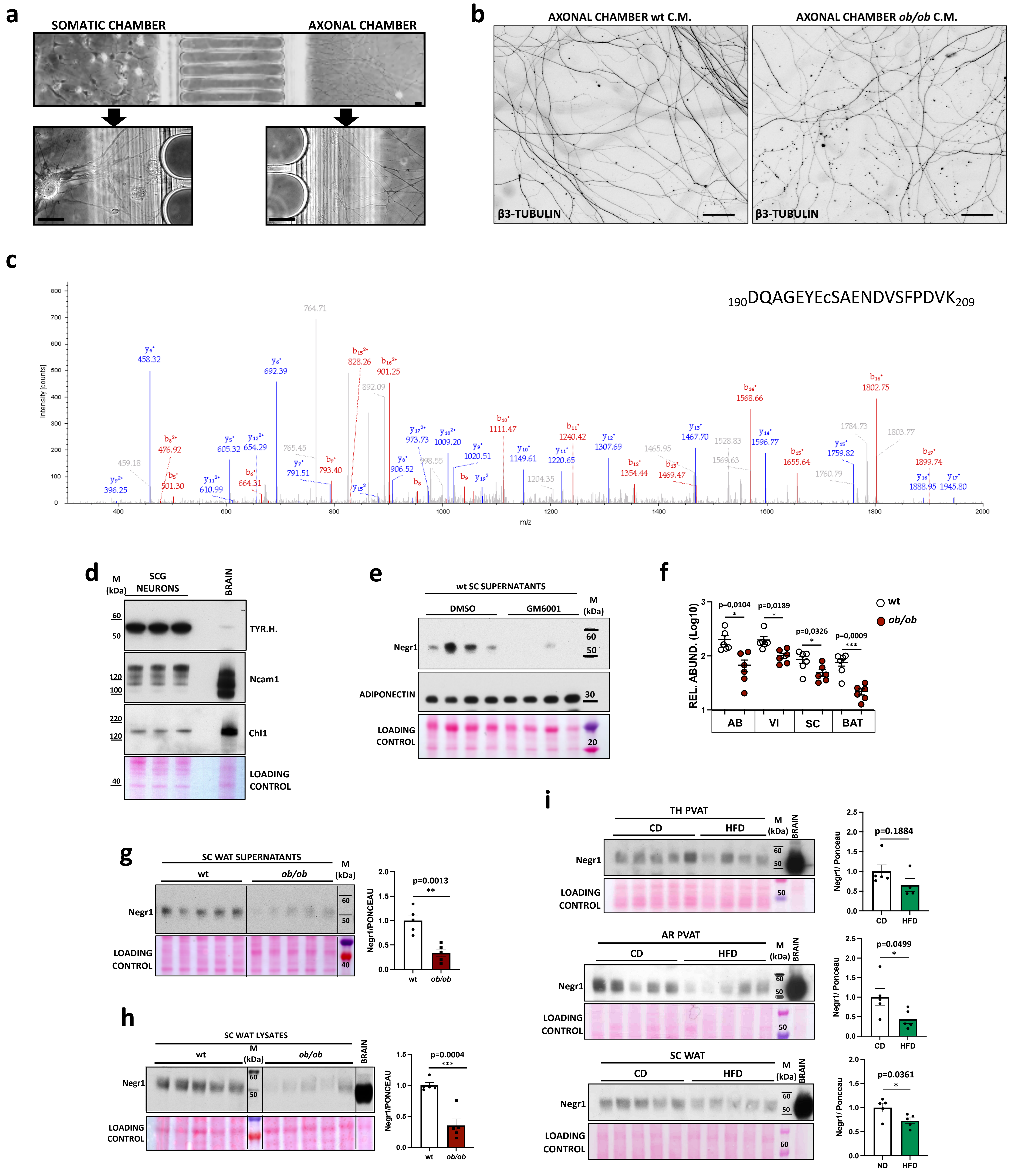

Supplementary Figure 4. Characterization of molecular mechanisms involved in obesity-induced neurodegeneration. a, Representative brightfield pictures of SCG sympathetic neurons cultured for 3 days in a microfluidic device in presence of 10 ng/ml NGF. Scale bars: 20 µm. **b,** Representative lower magnification pictures of

axonal chambers (1 out of 3 replicates) incubated for 24 h with either wt or *ob/ob* explants conditioned media (C.M.) and then stained for β3-tubulin. Scale bar: 100 µm. **c,** Representative MS/MS spectrum of peptide from murine Negr1 detected by mass spectrometry. **d,** Western blot showing cultured SCGs sympathetic neurons lysates from 3 biological replicates stained for sympathetic neuronal marker TYR.H. and for cell adhesion molecules Ncam1 and Chl1. Brain lysate was loaded as positive control. **e**, Western blot on supernatants from SC WAT explants from wt mice incubated for 24 h in absence (DMSO) or presence of the wide spectrum MMP inhibitor GM6001 and stained for Negr1 and Adiponectin. **f,** MS-determined relative abundances of Negr1 in the secretomes of wt and *ob/ob* AB PVAT, VI and SC WAT and BAT. N=6 biological replicates per group, unpaired, two-tailed, independent t-test. Mean ± SEM are shown. **g** and **h,** Western blot showing Negr1 staining in the conditioned media (**g**) and lysates (**h**) of wt and *ob/ob* SC WAT and relative quantifications. Data are shown as mean ± SEM, N=5 biological replicates, unpaired, two-tailed, independent t-test. **i**, Western blot and relative quantifications showing Negr1 abundancies in AR and TH PVAT and SC WAT of wt mice fed with a high-fat diet (HFD) or a control diet (CD) for 16 weeks. Data are shown as mean ± SEM, N=5 biological replicates per group except the HFD TH PVAT group (N=4), and analyzed by unpaired, two-tailed, independent t-test.

AB: abdominal PVAT; VI: visceral AT; SC: subcutaneous AT; BAT: interscapular brown AT. M: molecular weight marker. Negr1: neuronal growth regulator 1. TYR.H.: tyrosine hydroxylase; Ncam1: neural cell-adhesion molecule 1; Chl1: close homologue of L1. NGF: nerve growth factor. Source data are provided as a Source Data file.

Supplementary Figure 5

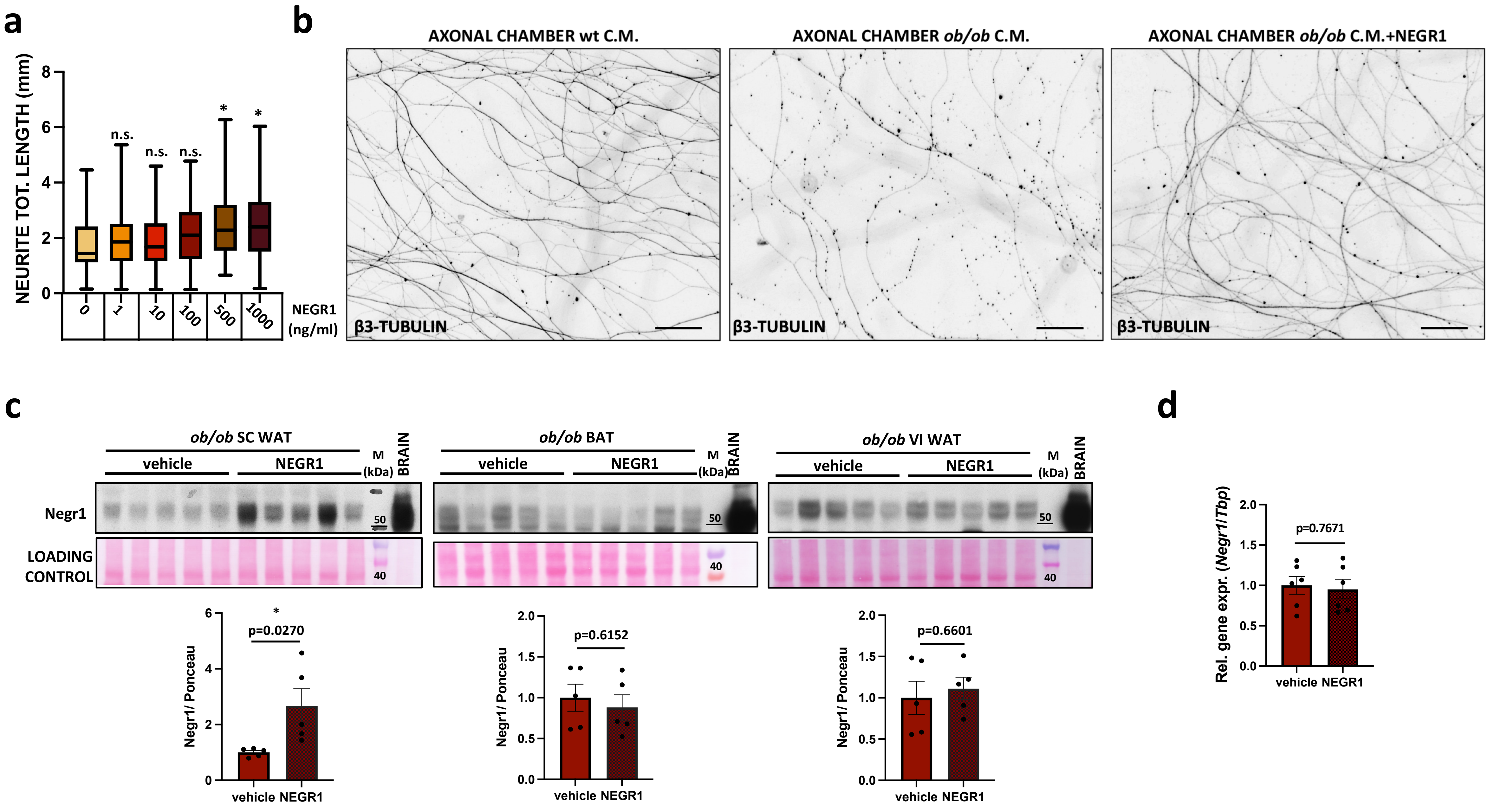

Supplementary Figure 5. Neurotrophic effect of Negr1 on sympathetic neurons. a, Quantification of total axonal length performed on primary sympathetic neurons incubated for 24 hours in medium supplemented with 1 ng/ml NGF and the indicated concentrations of Negr1. Box and whiskers plot shows median, quartiles and min. to max. values of 40<N<50 neurons per condition from 3 independent experiments. *p=0.0112 for NEGR1 500 ng/ml and p=0.0117 for NEGR1 1000 ng/ml by one-way ANOVA followed by Tuckey's multiple comparisons test. **b,** Representative lower magnification pictures of microfluidics axonal chambers (1 out of 3 replicates) incubated for 24 h with conditioned media (C.M.) from wt and *ob/ob* fat explants +/- recombinant Negr1 (500 ng/ml) and then stained for β3-tubulin. Scale bar: 100 µm. **c,** Western blots and relative quantifications showing Negr1 abundances in SC WAT, BAT and VI WAT of mice receiving for 2 weeks either recombinant Negr1 or saline at the level of SC WAT via catheter-connected minipumps. Data are shown as mean ± SEM, N=5 biological replicates per group, unpaired, two-tailed, independent t-test. **d,** Relative mRNA levels of *Negr1* normalized on *Tbp* in SC WAT lysates of mice receiving for 2 weeks either recombinant Negr1 or saline at the level of SC WAT via catheter-connected minipumps. Data are shown as mean ± SEM, N=5 biological replicates per group, unpaired, two-tailed, independent t-test.

VI WAT: visceral white AT; SC: subcutaneous WAT; BAT: interscapular brown AT. M: molecular weight marker. Negr1: neuronal growth regulator 1. Source data are provided as a Source Data file.

Supplementary Table 1

	(BMI≤30)	(BMI≥30)	p-value
**Patients (N)**	12	8	-
**Males (N)**	11	5	-
**Females (N)**	1	3	-
**Age**	58.2 ± 12.4	64.1 ± 12.8	0.3132
**Sex (%M)**	91.7%	62.5%	0.2553
**BMI**	26.1 ± 2.6	34.9 ± 6.7	0.0006
**BBK pre-op**	54.5%	55.5%	1.0000
**Hypertension**	63.6%	87.5%	0.3246
**Diabetes**	8.3%	75.0%	0.0044

Supplementary Table 1. Table reporting the characteristics of the 20 patients included in our study. Categorical data are shown as %, continuous data are shown as mean ± SD. P-values are calculated by Fisher's exact test for categorical data and by unpaired t-test for continuous data.

Uncropped blots Supplementary Figure 1b

ANTI-ADIPONECTIN

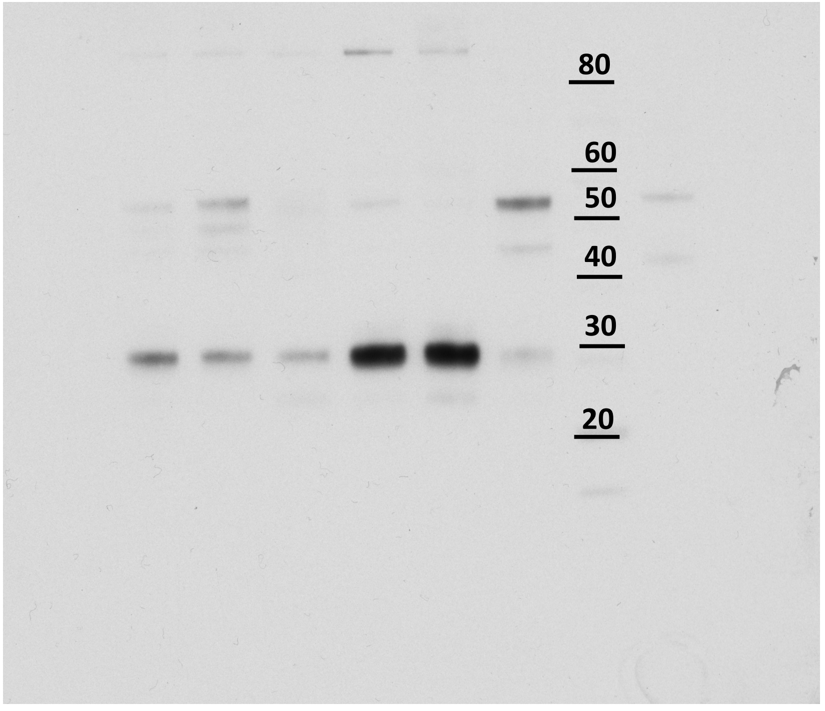

ANTI-PAI1

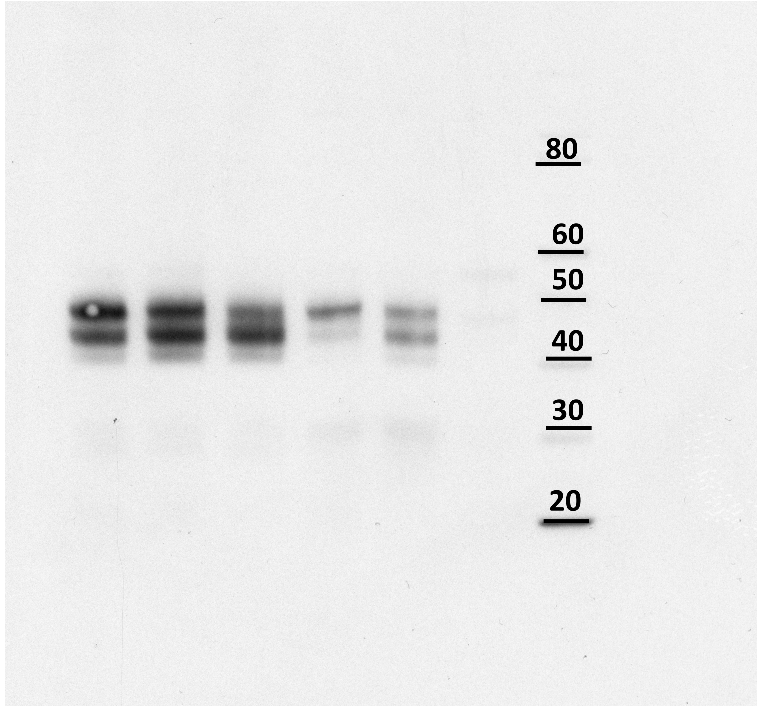

ANTI-VISFATIN

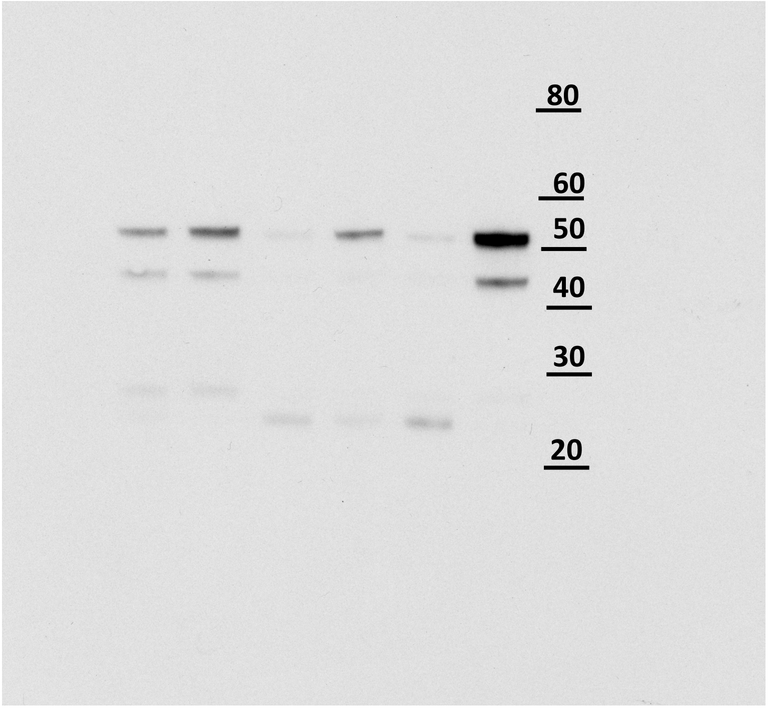

Uncropped blots Supplementary Figure 3a

ANTI-TYR.H.

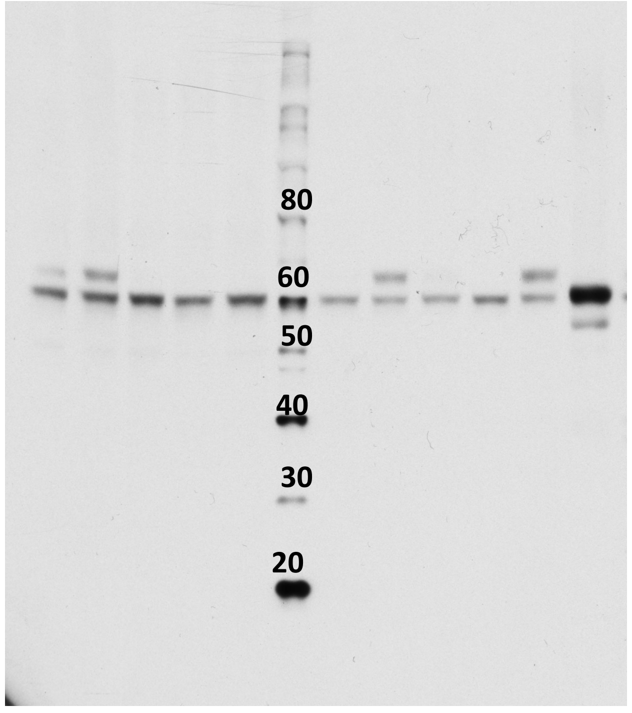

ANTI-UCP1

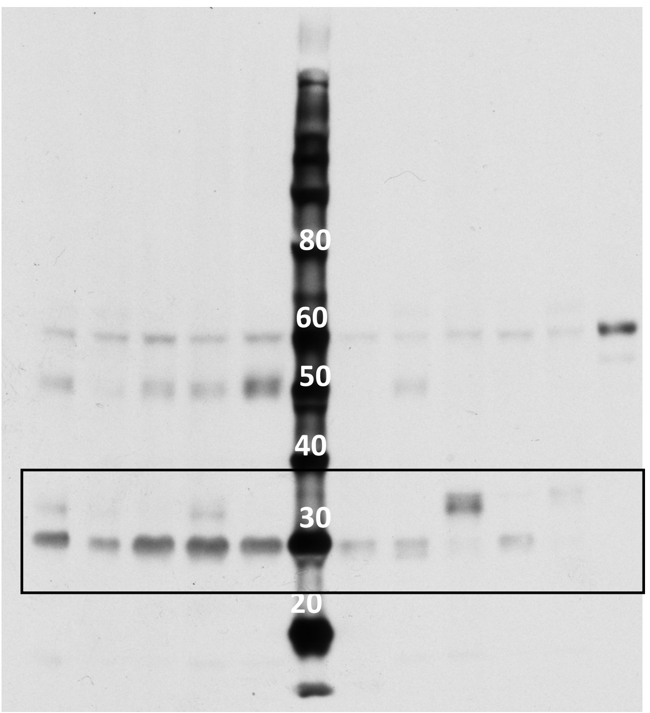

Uncropped blots Supplementary Figure 3b

ANTI-TYR.H.

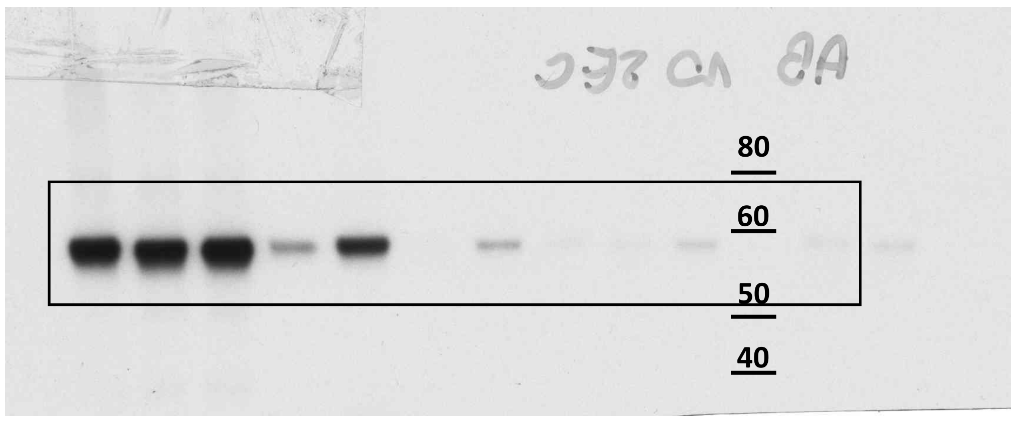

Uncropped blots Supplementary Figure 3c

ANTI-TYR.H.

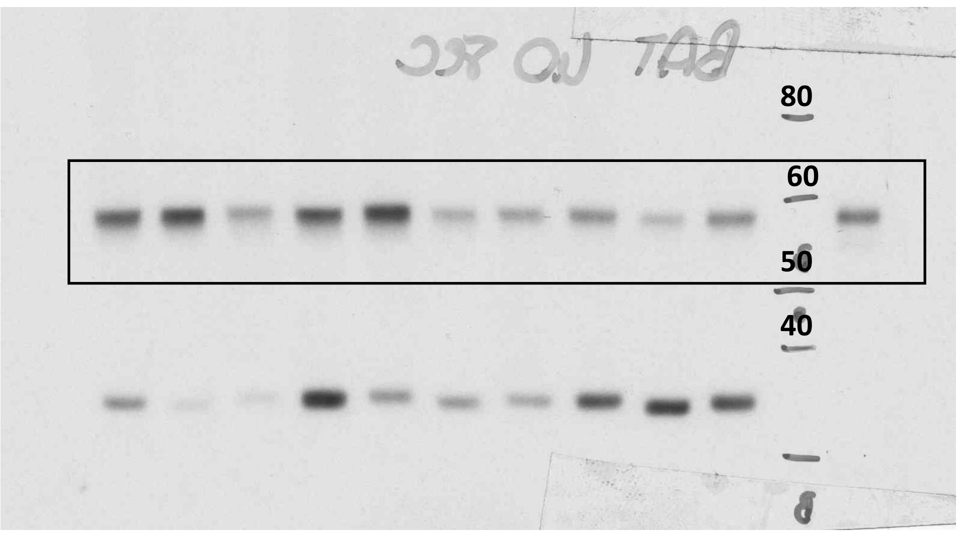

Uncropped blots Supplementary Figure 3d

ANTI-TYR.H. (AR PVAT)

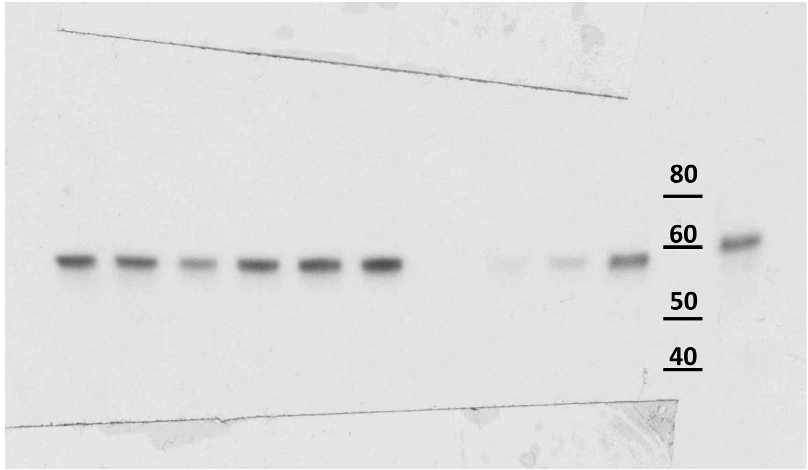

ANTI-TYR.H. (TH PVAT)

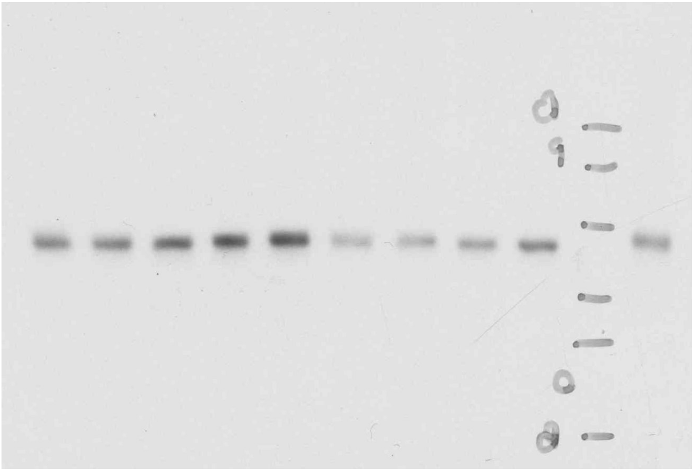

ANTI-TYR.H. (SC WAT)

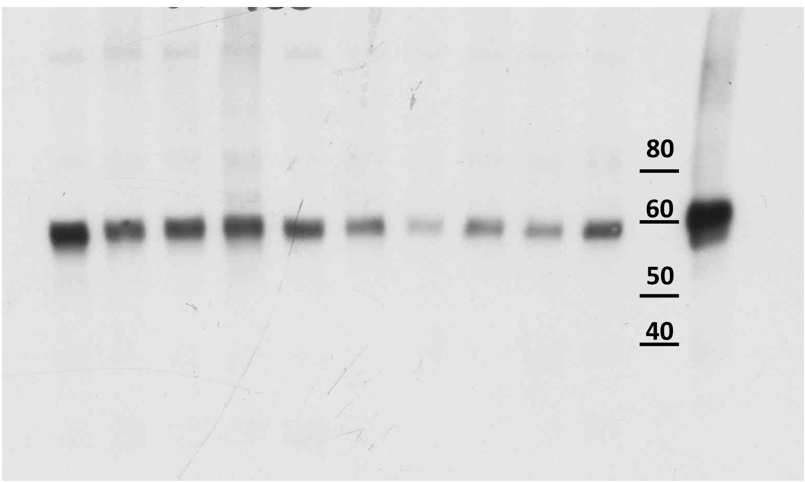

Uncropped blots Supplementary Figure 4d

ANTI-TYR.H.

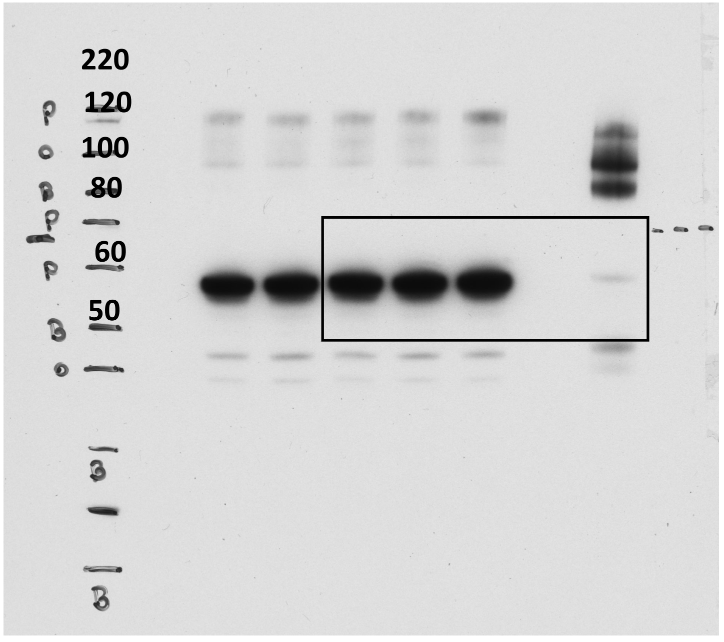

ANTI-CHL1

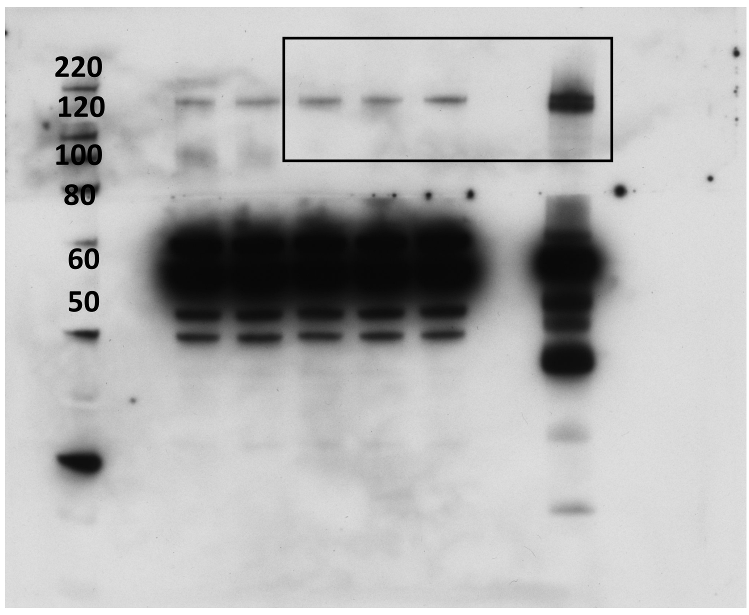

ANTI-NCAM1

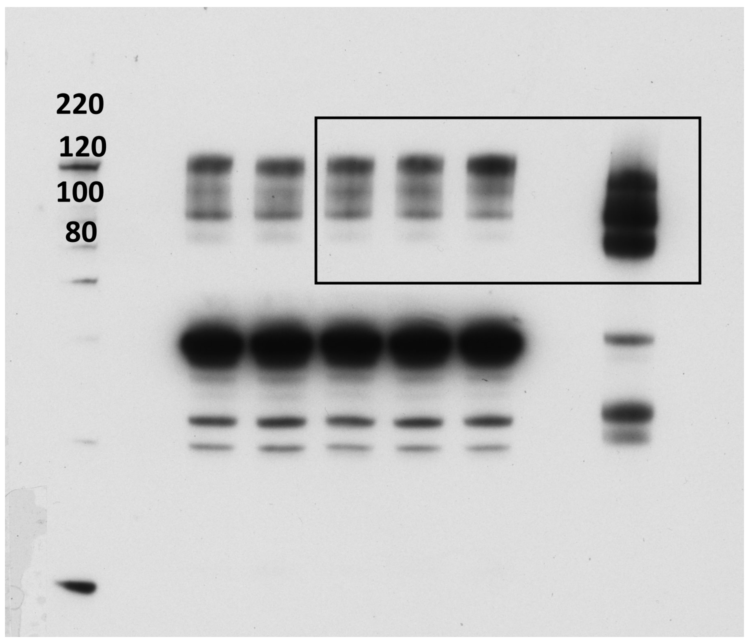

Uncropped blots Supplementary Figure 4e

ANTI-NEGR1

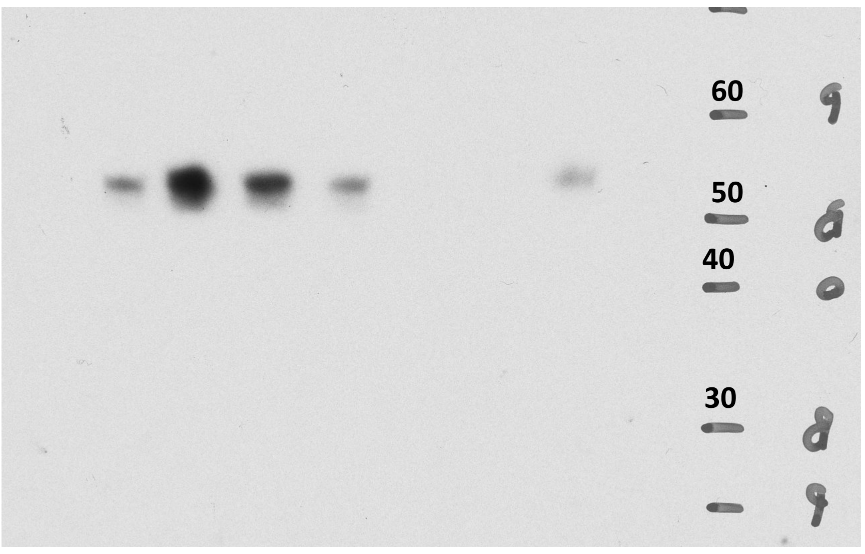

ANTI-ADIPONECTIN

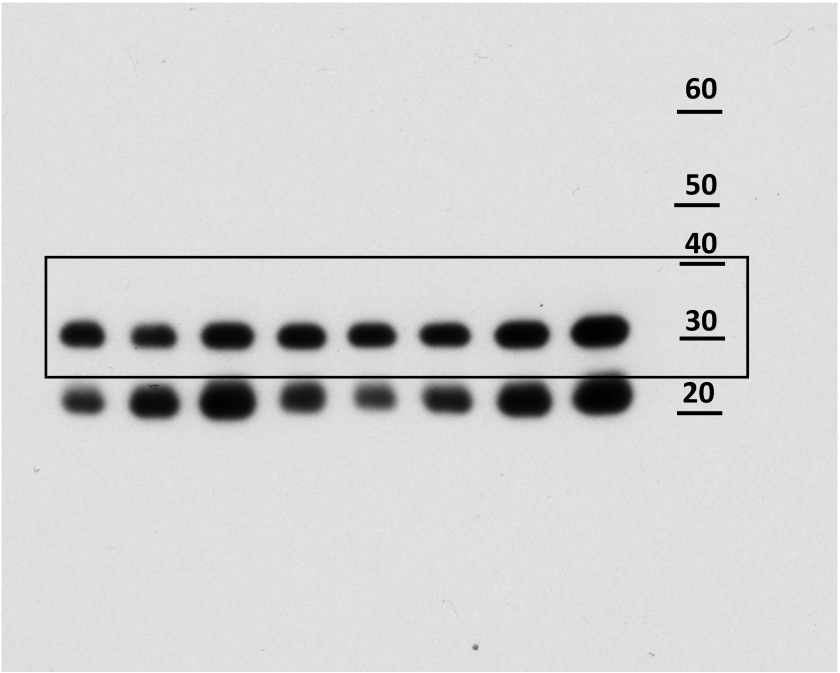

Uncropped blots Supplementary Figure 4g

ANTI-NEGR1

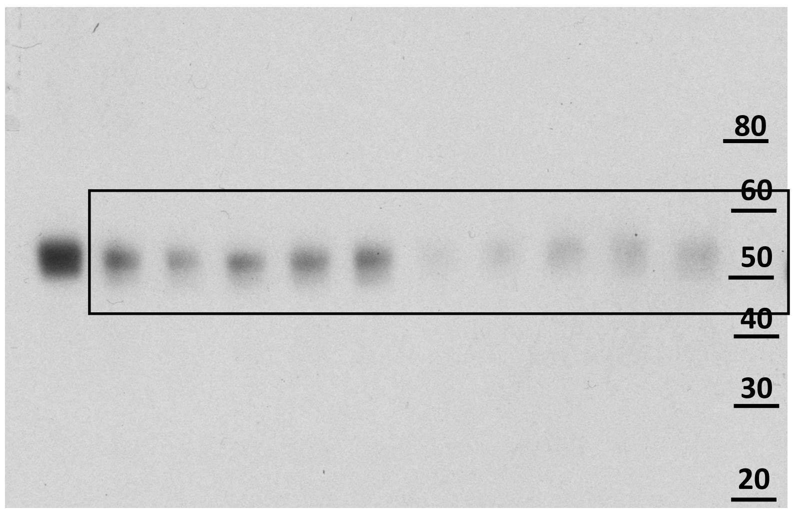

Uncropped blots Supplementary Figure 4h

ANTI-NEGR1

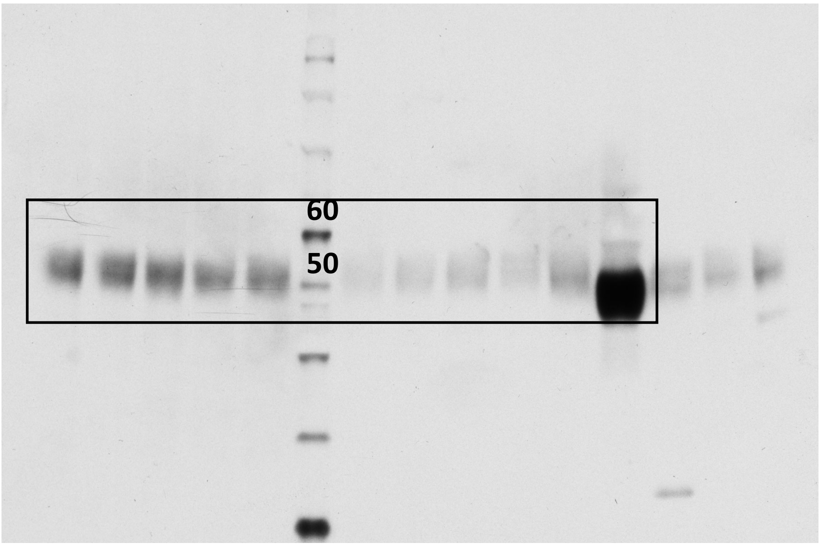

Uncropped blots Supplementary Figure 4i

ANTI-NEGR1 (AR PVAT)

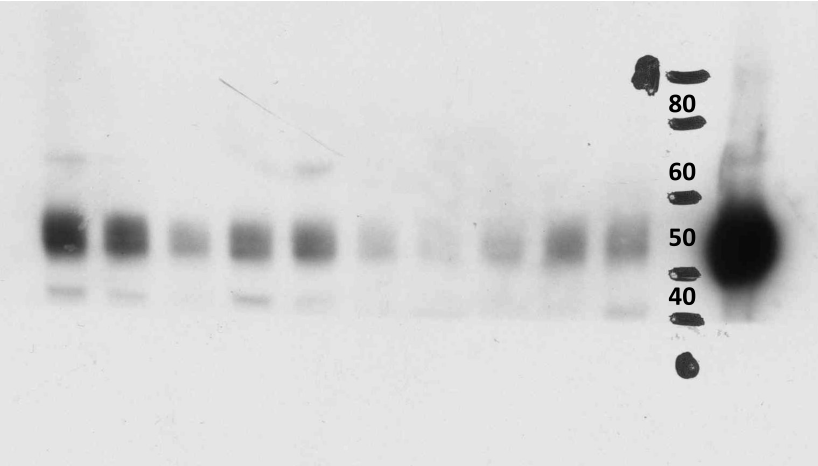

ANTI-NEGR1 (TH PVAT)

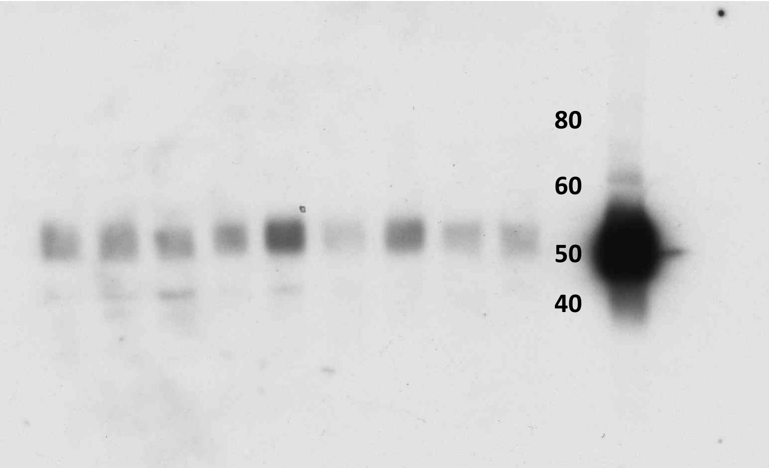

ANTI-NEGR1 (SC WAT)

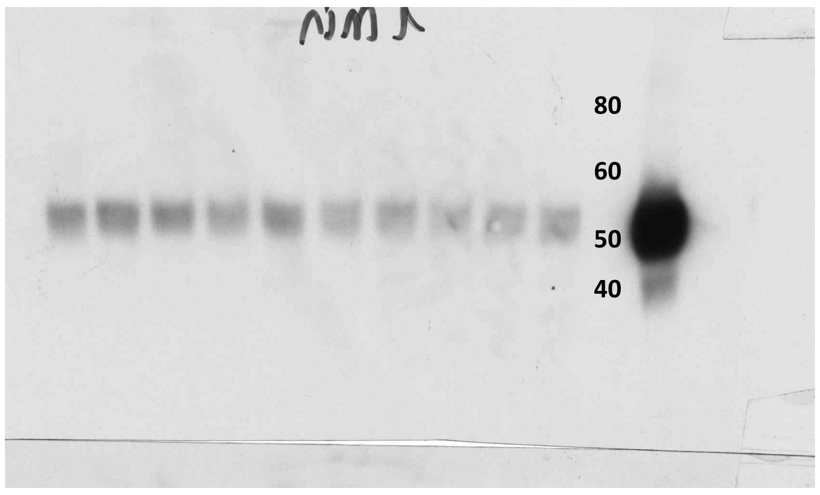

Uncropped blots Supplementary Figure 5c

NEGR1 SC

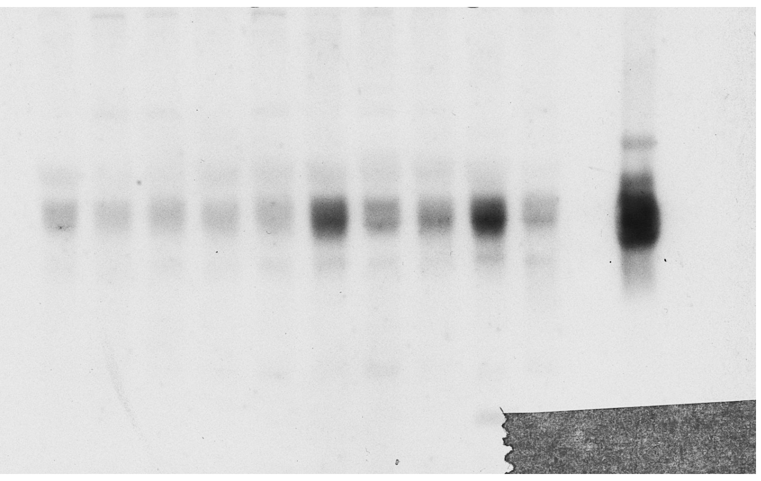

NEGR1 BAT

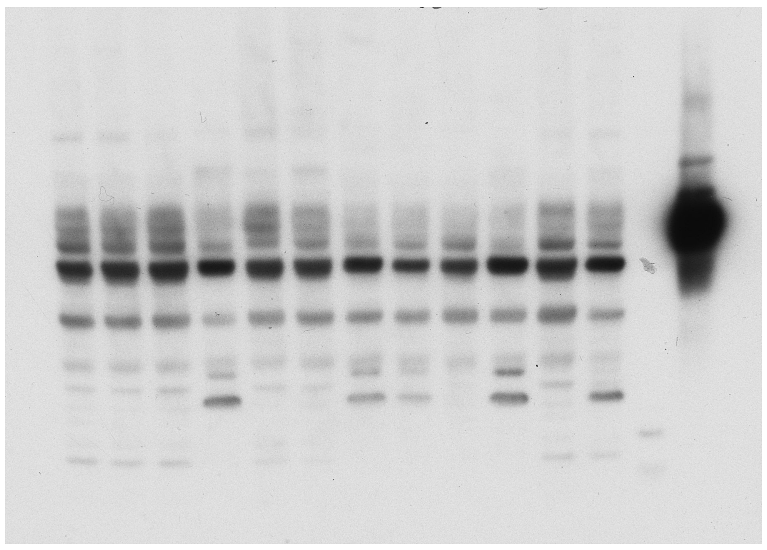

NEGR1 VI

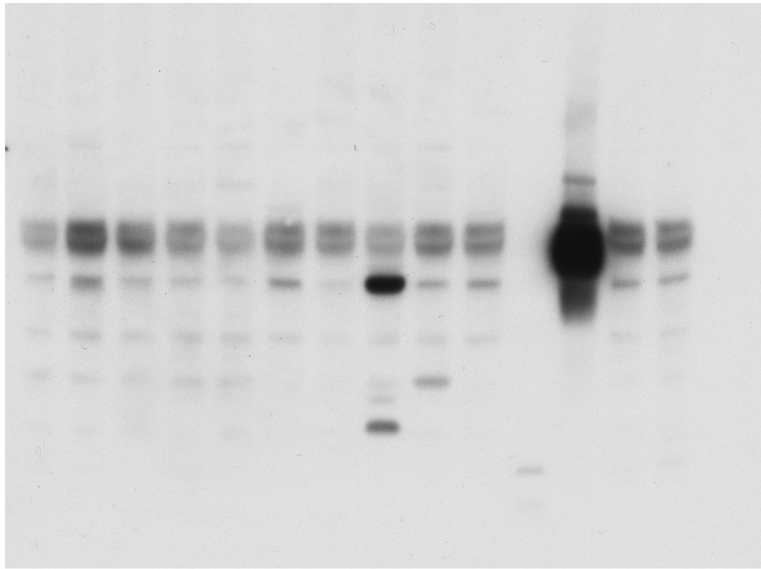

Supplement: Supplementary file 1 — Supplementary Information [file 41467_2022_34846_MOESM1_ESM.pdf]
