# Supplementary material for: Reduced secretion of neuronal growth regulator 1 contributes to impaired adipose-neuronal crosstalk in obesity
Source: Nat Commun. 2022 Nov 25;13:7269. doi: 10.1038/s41467-022-34846-w (PMC9700863; doi:10.1038/s41467-022-34846-w)
Supplement: Supplementary file 3 — Description of Additional Supplementary Files [file 41467_2022_34846_MOESM3_ESM.pdf]

Description of Additional Supplementary Files

File Name: Supplementary Movie 1
Description: **Tyrosine hydroxylase structural distribution in wt thoracic PVAT.** Movie shows progressive z-stacks through a 16 μm representative section of wt thoracic PVAT stained for tyrosine hydroxylase (in green) and perilipin (in red). Nuclei are counterstained with DAPI. Scale bar: 50 μm. One out of 4 replicates is shown.

File Name: Supplementary Movie 2
Description: **Tyrosine hydroxylase structural distribution in *ob/ob* thoracic PVAT.** Movie shows progressive z-stacks through a 16 μm representative section of *ob/ob* thoracic PVAT stained for tyrosine hydroxylase (in green) and perilipin (in red). Nuclei are counterstained with DAPI. Scale bar: 50 μm. One out of 4 replicates is shown.

File Name: Supplementary Data 1
Description: Proteins identified in conditioned media of AT depots from wt mice. Table shows: the 2407 non redundant proteins detected by untargeted LC-MS/MS in conditioned media of AT from wt mice, filtered for secretion according to SignalP, Matrisome and TargetP (2407 DETECTED); the statistical paired comparison between PVAT and non-PVAT secretory profiles (PVAT vs. NON-PVAT, N=18 vs. 18) and between each single depot and the remaining ones (BAT, SC, VI, AR, TH and AB vs. ALL OTHERS, N=6 vs. 30). The limma package was used to compare different groups using the Ebayes algorithm followed by Benjamini-Hochberg adjustment.

File Name: Supplementary Data 2
Description: Proteins identified in conditioned media of PVAT depots from wt and *ob/ob* mice. Table shows: the 1971 non redundant proteins detected by LC-MS/MS in conditioned media of PVAT depots from wt and *ob/ob* mice, filtered for secretion according to SignalP, Matrisome and TargetP (1971 DETECTED); the statistical unpaired comparison between wt and *ob/ob* PVAT secretomes (wtPVAT vs. obobPVAT, N=18 vs. 18). The limma package was used to compare different groups using the Ebayes algorithm followed by Benjamini-Hochberg adjustment.
