# Supplementary material for: Reduced secretion of neuronal growth regulator 1 contributes to impaired adipose-neuronal crosstalk in obesity
Source: Nat Commun. 2022 Nov 25;13:7269. doi: 10.1038/s41467-022-34846-w (PMC9700863; doi:10.1038/s41467-022-34846-w)
Supplement: Supplementary file 8 — Reporting Summary [file 41467_2022_34846_MOESM8_ESM.pdf]

nature portfolio

Corresponding author(s): Manuel Mayr

Last updated by author(s): October 11, 2022

Reporting Summary

Nature Portfolio wishes to improve the reproducibility of the work that we publish. This form provides structure for consistency and transparency in reporting. For further information on Nature Portfolio policies, see our Editorial Policies and the Editorial Policy Checklist.

Statistics

For all statistical analyses, confirm that the following items are present in the figure legend, table legend, main text, or Methods section.

n/a	Confirmed	
☐	☒	The exact sample size (*n*) for each experimental group/condition, given as a discrete number and unit of measurement
☐	☒	A statement on whether measurements were taken from distinct samples or whether the same sample was measured repeatedly
☐	☒	The statistical test(s) used AND whether they are one- or two-sided<br>*Only common tests should be described solely by name; describe more complex techniques in the Methods section.*
☒	☐	A description of all covariates tested
☐	☒	A description of any assumptions or corrections, such as tests of normality and adjustment for multiple comparisons
☐	☒	A full description of the statistical parameters including central tendency (e.g. means) or other basic estimates (e.g. regression coefficient) AND variation (e.g. standard deviation) or associated estimates of uncertainty (e.g. confidence intervals)
☐	☒	For null hypothesis testing, the test statistic (e.g. *F*, *t*, *r*) with confidence intervals, effect sizes, degrees of freedom and *P* value noted<br>*Give P values as exact values whenever suitable.*
☒	☐	For Bayesian analysis, information on the choice of priors and Markov chain Monte Carlo settings
☐	☒	For hierarchical and complex designs, identification of the appropriate level for tests and full reporting of outcomes
☒	☐	Estimates of effect sizes (e.g. Cohen's *d*, Pearson's *r*), indicating how they were calculated

Our web collection on statistics for biologists contains articles on many of the points above.

Software and code

Policy information about availability of computer code

Data collection	Nikon Elements (NIS-elements 4.0) was used to acquire images on a Nikon Spinning Disc confocal. LAS V4.3 software was used to acquire images on a Leica DM2000 microscope. LAS X (Leica Application Suite X, version 3.6.0.20104) was used to acquire images on a Leica DM IL LED inverted microscope. EPSON scan software (version 3.9.2.1) was used to scan Western blot films for subsequent analysis. LC-MS data were acquired using Tune (version 3.0, Thermo Scientific) and Xcalibur (version 4.1, Thermo Scientific). Proteome Discoverer (version 2.2.0.388, Thermo Scientific) linked to a Mascot server (version 2.6.0, Matrix Science) was used to search LC-MS data.
Data analysis	Fiji (ImageJ) software (version 2.0.0) was used to quantify Western blots and to quantify the structural axonal integrity. The NeuronJ plugin was used to analyse the arborization complexity of cultured neurons. ViiA 7 software (Applied Biosystems) was used to analysed qRNA data. GraphPad (Prism, version 8.01 and 9.2.0) was used to prepare some charts including bar charts, volcano plots, dot plots, violin and box and whiskers plots and to perform t-tests, one-way ANOVA tests and correlation analysis. The Ebayes method of the limma package was used for differential expression analysis in the proteomics data and corrections for multiple testing were conducted with the Benjamini-Hochberg method using python scripts. David bioinformatics tool (version 6.8) was used to perform biological processes functional enrichment analysis. Heatmaps and hierarchical clustering were performed using the ggplot2 package (version 2.2.1) of R environment (version 4.1.2). Principal component analysis was conducted using the scikit-learn python library (version 0.19.2) and 3D scatterplots were visualized using python the matplotlib library (version 3.5.1).

For manuscripts utilizing custom algorithms or software that are central to the research but not yet described in published literature, software must be made available to editors and reviewers. We strongly encourage code deposition in a community repository (e.g. GitHub). See the Nature Portfolio guidelines for submitting code & software for further information.

Data

Policy information about availability of data

All manuscripts must include a data availability statement. This statement should provide the following information, where applicable:

- Accession codes, unique identifiers, or web links for publicly available datasets
- A description of any restrictions on data availability
- For clinical datasets or third party data, please ensure that the statement adheres to our policy

LC-MS data were searched against the murine database UniProtKB/Swiss-Prot (version May 2018, https://ftp.uniprot.org/pub/databases/uniprot/previous_releases/release-2018_05/knowledgebase/uniprot_sprot-only2018_05.tar.gz). SignalP (version 4.1, https://services.healthtech.dtu.dk/service.php?SignalP-4.1), MatrisomeDB 2.0 (http://matrisomeproject.mit.edu) and TargetP (version 2.0, https://services.healthtech.dtu.dk/service.php?TargetP-2.0) were used to select secreted proteins. All data generated or analysed during this study are included in this published article and in its supplementary information files. Source Data are provided with this paper. The mass-spectrometry proteomics data generated and analysed during the current study have been deposited to the ProteomeXchange Consortium via the PRIDE partner repository with the dataset identifier PXD031271 and 10.6019/PXD031271 (https://doi.org/10.6019/PXD031271).

Field-specific reporting

Please select the one below that is the best fit for your research. If you are not sure, read the appropriate sections before making your selection.

☒ Life sciences ☐ Behavioural & social sciences ☐ Ecological, evolutionary & environmental sciences

For a reference copy of the document with all sections, see nature.com/documents/nr-reporting-summary-flat.pdf

Life sciences study design

All studies must disclose on these points even when the disclosure is negative.

Sample size	Sample size for every experiment is reported in the Figure legend or in the Methods section.<br>For quantitative analyses, sample size was determined based on similar studies in the same field and no power/sample size calculation was performed.<br>For each in vitro experiment, at least 3 biological replicates were performed based on articles published on relevant journals and performing similar experiments (see ref. 32 and 45 in the manuscript as well as the following articles: https://pubmed.ncbi.nlm.nih.gov/20348920/, https://pubmed.ncbi.nlm.nih.gov/18701687/).<br>For animal experiments, 4 to 6 animals were used per group as similar N numbers have been employed in similar studies published in high impact factor journals (see ref. 25 and 30 in the manuscript). N numbers employed here were sufficient to demonstrate significant/clear cut differences in the compared groups within each experiment.<br>The number of human samples used in this study was determined by samples availability in this time window.
Data exclusions	One out of 6 points was excluded from the dot plot in Supplementary Fig. 2b as also reported in the Source Data file. This point was identified as an outlier based on the ROUT method, Q=1% (GraphPad, Prism).<br>In vitro experiments on microfluidics were only performed when seeding and axonal growth were successful.<br>No other data were excluded from analysis.
Replication	To ensure reproducibility, all in vitro experiments on primary sympathetic neurons were repeated at least 3 times. A distinct litter/group of litters was used in every biological replicate. All attempts at replication were successful. N size for quantitative animal experiments was always 4 to 6 mice per condition. Histology experiments that did not require quantification (such as H&E staining or neuronal proteins localization) were repeated at least 2 times and similar outcomes were observed in all replicates. The same applies for some Western blot experiments that were not quantified as they were performed to provide a qualitative rather than quantitative information, such as those in fig. 1b, 1c, 6d, 6e and 6f. All qualitative non-quantified experiments did provide clear-cut results. Western blots on pooled samples were only performed 1 time as 5 or 6 biological replicates were included in each pool. In some circumstances single samples were also run to verify the significance of the differences observed in the corresponding pools (such as tyrosine hydroxylase content in PVAT samples from wt and ob/ob mice in fig. 4a and 4b). N numbers are reported for all experiments in figure legends.
Randomization	For animal experiments comparing wt and ob/ob transgenic mice no randomization was used as the study design intentionally compared groups defined based on their different genotype. For experiments where animals with the same genotype were subjected to different treatments, mice were randomly assigned to the treated or control groups. Sex- and age-matched mice were always used within each experiment. For experiments involving human patients, subjects were grouped based on their BMI ($\leq$30 or $\geq$30) for the comparison between patients without and with obesity. Different grouping were made to test the effect of relevant covariates such as sex (male and female groups) and pre-op. beta-blockers administration (administered or not administered). For in vitro experiments, neurons from the same culture seeded in distinct wells on the same plate or in distinct microfluidic devices were randomly allocated to different treatments.
Blinding	For the quantification of axonal swelling, images acquisition and analysis were performed in blind. The quantification of axonal elongation and branching was not performed in blind. This is because neurons were seeded at such a low density that all neurons in each coverslip were acquired and analysed in every tested condition (meaning that no neurons were excluded from the anlysis). Also, quantification of axonal elongation and branching are objective measurements that are not subject to personal interpretation. The analysis of axonal fragmentation in perivascular fat sections was not performed in blind as samples belonging to ob/ob animals were clearly recognizable due to adipocyte hypertrophy highlighted by perilipin staining.

Reporting for specific materials, systems and methods

We require information from authors about some types of materials, experimental systems and methods used in many studies. Here, indicate whether each material, system or method listed is relevant to your study. If you are not sure if a list item applies to your research, read the appropriate section before selecting a response.

Materials & experimental systems

n/a	Involved in the study
☐	☒ Antibodies
☒	☐ Eukaryotic cell lines
☒	☐ Palaeontology and archaeology
☐	☒ Animals and other organisms
☐	☒ Human research participants
☒	☐ Clinical data
☒	☐ Dual use research of concern

Methods

n/a	Involved in the study
☒	☐ ChIP-seq
☒	☐ Flow cytometry
☒	☐ MRI-based neuroimaging

Antibodies

Antibodies used

Primary antibodies:
Anti-Perilipin-1 antibody, Abcam, ab61682, Goat polyclonal to Mouse and Human Perilipin-1
Anri-UCP1 antibody, Abcam, ab23841, Rabbit polyclonal to Mouse, Rat and Dog UCP1
Anti-beta 3 Adrenergic Receptor antibody, Abcam, ab94506, Rabbit polyclonal to Mouse beta 3 Adrenergic Receptor
Anti NCAM1 antibody, Abcam, ab220360, Rabbit monoclonal to Mouse and Rat NCAM1
Anti-beta III Tubulin antibody, Abcam, ab18207, Rabbit polyclonal to Mouse, Rat, Human, Pig, etc. beta III Tubulin
Anti-L1CAM antibody, Abcam, ab208155, Rabbit monoclonal to Mouse, Rat, Human L1CAM
Anti-Adiponectin antibody, Abcam, ab22554, Mouse monoclonal to Mouse, Rat, Rabbit, Human, Baboon Adiponectin
Anti Serpin E1/PAI-1 antibody, R&D SYSTEMS, AF3828, Goat polyclonal to Mouse PAI-1
Anti PBEF/Visfatin antibody, R&D SYSTEMS, MAB40441, clone # 882104, Mouse monoclonal to human Visfatin
Anti CHL-1/L1CAM-2 antibody, R&D SYSTEMS, AF2147, Goat polyclonal to mouse CHL-1
Anti Kilon/NEGR1 antibody, R&D SYSTEMS, AF5394, Goat polyclonal to Human and Mouse NEGR1
Anti-Tyrosine Hydroxylase antibody, Millipore, AB152, Rabbit polyclonal to Mouse, Rat, Human Tyrosine Hydroxylase
Anti-Tyrosine Hydroxylase, Millipore antibody, AB1542, Sheep polyclonal to Mouse, Rat Tyrosine Hydroxylase
Anti-beta Actin antibody, Sigma-Aldrich, A1978, Mouse monoclonal to Human, Bovine, Sheep, Pig, Rabbit, Cat, Dog, Mouse, Rat beta Actin

Secondary antibodies:
Peroxidase IgG Fraction Monoclonal Mouse Anti-Goat IgG, light chain specific, Jackson, 205-032-176
Peroxidase IgG Fraction Monoclonal Mouse Anti-Rabbit IgG, light chain specific, Jackson, 211-032-171
Peroxidase AffinityPure Goat Anti-Mouse IgG, light chain specific, Jackson, 115-035-174
Sheep IgG HRP-conjugated Antibody, R&D SYSTEMS, HAF016
Donkey anti-Goat IgG (H+L) Cross-Adsorbed Secondary Antibody, Alexa Fluor 647, ThermoFisher, A-2147
Donkey anti-Rabbit IgG (H+L) Highly Cross-Adsorbed Secondary Antibody, Alexa Fluor 594, A-21027
Donkey anti-Goat IgG (H+L) Cross-Adsorbed Secondary Antibody, Alexa Fluor 488, A-11055
Donkey anti-Rabbit IgG (H+L) Highly Cross-Adsorbed Secondary Antibody, Alexa Fluor 647, A-31573

Validation

All primary antibodies were chosen based on validation information on the manufacturer's website. In addition, all antibodies against neuronal proteins (NCAM1, L1CAM, beta III Tubulin, CHL-1, NEGR1 and Tyrosine Hydroxylase) were validated in murine brain lysates, and positive controls were included in Western blots when possible.
Information about validation in different techniques can be found at the following links:
https://www.abcam.com/perilipin-1-antibody-ab61682.html
https://www.abcam.com/ucp1-antibody-ab23841.html
https://www.abcam.com/beta-3-adrenergic-receptor-antibody-ab94506.html
https://www.abcam.com/ncam1-antibody-epr21827-ab220360.html
https://www.abcam.com/beta-iii-tubulin-antibody-neuronal-marker-ab18207.html
https://www.abcam.com/l1cam-antibody-epr18750-ab208155.html
https://www.abcam.com/adiponectin-antibody-19f1-ab22554.html
https://www.rndsystems.com/products/mouse-serpin-e1-pai-1-antibody_af3828
https://www.rndsystems.com/products/human-mouse-rat-pbef-visfatin-antibody-882104_mab40441
https://www.rndsystems.com/products/mouse-chl-1-l1cam-2-antibody_af2147
https://www.rndsystems.com/products/human-mouse-kilon-negr1-antibody_af5394
https://www.merckmillipore.com/GB/en/product/Anti-Tyrosine-Hydroxylase-Antibody,MM_NF-AB152
https://www.merckmillipore.com/GB/en/product/Anti-Tyrosine-Hydroxylase-Antibody,MM_NF-AB1542
https://www.sigmaaldrich.com/GB/en/product/sigma/a1978

Animals and other organisms

Policy information about studies involving animals; ARRIVE guidelines recommended for reporting animal research

Laboratory animals	Wild type C57BL/6J male mice (12 or 18 weeks old) and age-matched male ob/ob mice (B6.Cg-Lepob/J, 10 weeks old for osmotic minipumps experiment) were purchased from Charles River.<br>For HFD experiments, 5 weeks old wt C57BL/6 male mice were purchased from Janvier Labs and experiments were performed at Institute for Pharmacology and Clinical Phamacology, Düsseldorf, Germany.
Wild animals	This study did not involve wild animals.
Field-collected samples	This study did not involve samples collected in the field.
Ethics oversight	All animal procedures in the paper were performed in accordance with either i) the Guidance on the Operation of the Animals (Scientific Procedures) Act, 1986 (United Kingdom) with ethical approval obtained from King's College London Committee for the Review of Ethics and Welfare (Licence number: 70/0843, Prof. Qingbo Xu and PP2172591, Dr. Ursula Mayr, approved by the Home Office under the Establishment license number X24D82DFF hold by King's College London); or ii) the guidelines for the use of experimental animals as given by "Deutsches Tierschuntzgesetz" with ethical approval from the local Research Board for animal experimentation (State Agency for Nature, Environment and Consumer Protection, file ref. 81-02.04.2017.A458).

Note that full information on the approval of the study protocol must also be provided in the manuscript.

Human research participants

Policy information about studies involving human research participants

Population characteristics	Epicardial fat samples employed in this study were collected from patients undergoing a first time isolated coronary artery bypass graft (CABG) surgery or aortic valve replacement at St. George's Hospital in London (UK) in 2012-2013 (REC reference: 12/LO/0422). Male and female patients were included in the study, with ages ranging from 36 to 85 years (see Source data file and Supplementary Fig. 3e for more detailed information). Exclusion criteria included: not adequate understanding of the study and consent; antiarrhythmic therapy other than beta blockers; presence of temporary or permanent pacemaker; off-pump-CABG surgery; systemic inflammatory disease requiring immunomodulating therapy; recent steroid therapy; HIV infection; lipodistrophy.
Recruitment	Eligible potential participants were identified by review of medical notes and approached upon admission. A patient information sheet containing all the relevant information regarding the study was provided and explained and all question were answered. Patients who agreed to participate were given at least 24 hours to decide whether or not to take part and were asked to sign a written informed consent form. A signed copy of the Informed Consent Form was given to the study participants along with the Patient Information Sheet. Participants did not receive any compensation.
Ethics oversight	Epicardial adipose tissue samples were collected from patients undergoing isolated coronary artery bypass graft surgery or aortic valve replacement at St. George's Hospital in London (UK) in 2012-2013 (Research Ethic Committee reference for ethical approval: 12/LO/0422, St. George's R&D approval: 12.0019). All subjects gave written informed consent before taking part in the study.

Note that full information on the approval of the study protocol must also be provided in the manuscript.
